# Supplementary material for: The revised three-step detour pathway in dolichol biosynthesis is evolutionarily conserved in budding yeast
Source: Proc Natl Acad Sci U S A. 2026 May 27;123(22):e2613147123. doi: 10.1073/pnas.2613147123 (PMC13229312; doi:10.1073/pnas.2613147123)
Supplement: Supplementary file 1 — Appendix 01 (PDF) [file pnas.2613147123.sapp.pdf]

PNAS

Supporting Information for

The revised three-step detour pathway in dolichol biosynthesis is evolutionarily conserved in budding yeast

Kazuki Hanaoka[1,5], Kuya Matsunaga[1,5], Souichirou Shimizu[1], Soshi Sakai[2], Harald Pichler[3,4], Kouichi Funato[1],[2]

1 Graduate School of Integrated Sciences for Life, Hiroshima University, Higashi-Hiroshima, 739-8528, Japan
2 School of Applied Biological Science, Hiroshima University, Higashi-Hiroshima, 739-8528, Japan
3 Institute of Molecular Biotechnology, Graz University of Technology, NAWI Graz, BioTechMed Graz, Petersgasse 14, 8010 Graz, Austria
4 Austrian Centre of Industrial Biotechnology (acib) GmbH, Petersgasse 14, 8010 Graz, Austria
5 These authors have contributed equally to this work.

* Corresponding author; Kouichi Funato

Email: kfunato@hiroshima-u.ac.jp

This PDF file includes:

Extended Materials and Methods
SI References

Extended Materials and Methods

Lists of the strains

Strain No.	Description	Genotype	Source	Corresponding Figure No.
FKY7486	WT	Mat a, ura3 his3 leu2 met15 lys2	This study	Fig. 1, 2
FKY8188	*ifa38*Δ	ura3 his3 leu2 *ifa38*Δ;;KanMX	This study	Fig. 1
FKY7195	*ayr1*Δ	Mat a, ura3 his3 leu2 lys2 MET15 *ayr1*Δ;;KanMX	This study	Fig. 1
FKY7236	*fox2*Δ	Mat a, ura3 his3 leu2 LYS2 met15 *fox2*Δ;;KanMX	This study	Fig. 1
FKY7256	*irc24*Δ	Mat a, ura3 his3 leu2 LYS2 met15 *irc24*Δ;;KanMX	This study	Fig. 1
FKY7274	*nre1*Δ	Mat a, ura3 his3 leu2 lys2 met15 *nre1*Δ;;KanMX	This study	Fig. 1
FKY7289	*oar1*Δ	Mat a, ura3 his3 leu2 LYS2 MET15 *oar1*Δ;;KanMX	This study	Fig. 1
FKY7304	*osi1*Δ	Mat alpha, ura3 his3 leu2 lys2 met15 *osi1*Δ;;KanMX	This study	Fig. 1
FKY7320	*srl4*Δ	Mat a, ura3 his3 leu2 lys2 met15 *srl4*Δ;;KanMX	This study	Fig. 1
FKY7339	*sps19*Δ	Mat alpha, ura3 his3 leu2 lys2 met15 *sps19*Δ;;KanMX	This study	Fig. 1
FKY7356	*ykl107w*Δ	Mat a, ura3 his3 leu2 LYS2 MET15 *ykl107w*Δ;;KanMX	This study	Fig. 1
FKY7513	*tda5*Δ	Mat a, ura3 his3 leu2 MET15 lys2 *tda5*Δ;;KanMX	This study	Fig. 1, 2
FKY7630	*env9*Δ	Mat alpha, ura3 his3 leu2 MET15 lys2 *env9*Δ;;KanMX	This study	Fig. 1, 2
FKY7638	*ydl114w*Δ	ura3 his3 leu2 MET15 lys2 *ydl114w*Δ;;KanMX	This study	Fig. 1
FKY7988	*dfg10*Δ	Mat a, ura3 his3 leu2 MET15 *dfg10*Δ;;KanMX	This study	Fig. 1, 2
FKY8091	WT	ura3 his3 leu2	This study	Fig. 1, 2
FKY8092	*tda5*Δ	ura3 his3 leu2 *tda5*Δ;;KanMX	This study	Fig. 1, 2
FKY8093	*dfg10*Δ	ura3 his3 leu2 *dfg10*Δ;;KanMX	This study	Fig. 1, 2
FKY8094	*tda5*Δ*dfg10*Δ	ura3 his3 leu2 *tda5*Δ;;KanMX *dfg10*Δ;;KanMX	This study	Fig. 1, 2

Lists of the plasmids

Plasmid No.	Name	Marker	Promoter	Source	Description	Correspon ding Figure
FKP 22	pRS416-GPD	URA3	GPD	Mumberg et al., Gene, 1995	empty	Fig. 1, 2

FKP 1234	pRS416-GPD *TDA5*	URA3	GPD	This study	The *TDA5* coding region was PCR-amplified from yeast genomic DNA and ligated into the BamHI-XhoI sites.	Fig. 1, 2
FKP 1278	pRS416-GPD *DFG10*	URA3	GPD	This study	The *DFG10* coding region was PCR-amplified from yeast genomic DNA and ligated into the BamHI-XhoI sites.	Fig. 1
FKP 1303	pRS416-GPD DHRSX	URA3	GPD	This study	The *DHRSX* coding region was PCR-amplified from human cDNA library and ligated into the BamHI-XhoI sites.	Fig. 1, 2
FKP 1315	pRS416-GPD SRD5A3	URA3	GPD	This study	The *SRD5A3* coding region was PCR-amplified from human cDNA library and ligated into the XbaI-XhoI sites.	Fig. 1, 2
FKP 1366	pRS416-GPD *ENV9*	URA3	GPD	This study	The *ENV9* coding region was PCR-amplified from yeast genomic DNA and ligated into the BamHI-XhoI sites.	Fig. 1

Construction of Gene Deletion Mutants

Gene deletion mutants were generated by transforming a PCR-amplified KanMX cassette into diploid BY wild-type cells, followed by homologous recombination. Haploid single mutants were isolated by tetrad dissection after sporulation, and gene deletions were confirmed by colony PCR. Double mutants were constructed by crossing the corresponding haploid single mutants, followed by sporulation, tetrad dissection, and colony PCR verification.

Serial dilution assays for tunicamycin sensitivity

Yeast cells, without or with plasmids, were cultured in rich YP medium (1% yeast extract, 2% peptone) supplemented with 0.2% adenine and containing 2% glucose (YPD) as carbon source at 25℃, and adjusted to OD_{600} 1.0 in sterile water. Fivefold serial dilutions were spotted onto fresh YPD plates containing tunicamycin at the concentrations indicated in the figure legends and incubated at 25℃ for 3 days.

Immunoblot analysis of CPY glycosylation

Yeast cells were grown in YPD at 25℃ to early log phase and lysed with glass beads in SDS-containing buffer. After removal of cell debris, lysates were treated with or without Endo-H at 37℃ for 60min, separated by SDS/PAGE, transferred to nitrocellulose membranes, and analyzed by immunoblotting with anti-CPY antibody. Signals were detected using HRP-conjugated secondary antibody and ECL substrate (1). Endo-H treatment was performed as described previously (2).

TLC analysis of neutral lipids and polyprenol/dolichol

Yeast cells were grown in YPD at 25℃ to early log phase, collected, washed, and adjusted to an OD_{600} of 10. Total lipids were extracted using chloroform-methanol-water (CMW, 10:10:3, v/v/v). For analysis of neutral lipids, extracts were separated by thin-layer chromatography (TLC) using petroleum ether-diethyl ether-acetic acid (25:25:1, v/v/v) for the first third of the plate, followed by petroleum ether-diethyl ether (49:1, v/v) for the remaining distance. Lipids were visualized by staining with MnCl2 reagent [0.63 g $MnCl_2 \cdot 4H_2O$ dissolved in methanol-water-sulfuric acid (60:60:4, v/v/v)] for 10 s (3,4). For analysis of polyprenol and dolichol, lipid extracts were separated by TLC using benzene-ethyl acetate (95:5, v/v) and visualized by exposure to iodine vapor for 15 min (5). Lipid bands were quantified using ImageJ (version 2.16.0/1.54p).

Statistical analysis

All statistical analyses and graphing were performed using R (R Foundation for Statistical Computing, Vienna, Austria). Comparisons among multiple groups were performed by one-way ANOVA followed by Tukey's multiple comparison test, as indicated in the figure legends. Data are presented as mean ± S.D. unless otherwise stated. In the graphs, bars indicate the mean, error bars indicate S.D., and dots indicate values from three independent biological replicates. Statistical significance is indicated as ns, not significant; *, $p < 0.05$; **, $p < 0.01$; ***, $p < 0.001$.

SI References

1. K. Kajiwara et al., Osh proteins regulate COPII-mediated vesicular transport of ceramide from the endoplasmic reticulum in budding yeast. *J. Cell Sci.* 127, 376–387 (2014).
2. N. Kageyama-Yahara, H. Riezman, Transmembrane topology of ceramide synthase in yeast. *Biochem. J.* 398, 585–593 (2006).
3. A. Ikeda et al., Tricalbins are required for non-vesicular ceramide transport at ER-Golgi contacts and modulate lipid droplet biogenesis. *iScience* 23, 101603 (2020).
4. Y. Yang et al., Retrograde Golgi-to-ER transport is regulated by diacylglycerol in Saccharomyces cerevisiae. *J. Cell Sci.* jcs264537 (2026).
5. M. Sato et al., The yeast *RER2* gene, identified by endoplasmic reticulum protein localization mutations, encodes cis-prenyltransferase, a key enzyme in dolichol synthesis. *Mol. Cell. Biol.* 19, 471–483 (1999).
